# Functional analysis of *RRAS2* pathogenic variants with a Noonan-like phenotype

**DOI:** 10.3389/fgene.2024.1383176

**Published:** 2024-03-27

**Authors:** Takaya Iida, Arisa Igarashi, Kae Fukunaga, Taiga Aoki, Tomomi Hidai, Kumiko Yanagi, Masahiko Yamamori, Kazuhito Satou, Hayato Go, Tomoki Kosho, Ryuto Maki, Takashi Suzuki, Yohei Nitta, Atsushi Sugie, Yoichi Asaoka, Makoto Furutani-Seiki, Tetsuaki Kimura, Yoichi Matsubara, Tadashi Kaname

**Affiliations:** ^1^ Department of Genome Medicine, National Center for Child Health and Development, Tokyo, Japan; ^2^ Department of Systems Biochemistry in Pathology and Regeneration, Yamaguchi University Graduate School of Medicine, Yamaguchi, Japan; ^3^ Department of Pediatrics, Fukushima Medical University School of Medicine, Fukushima, Japan; ^4^ Department of Medical Genetics, Shinshu University School of Medicine, Matsumoto, Japan; ^5^ School of Life Science and Technology, Tokyo Institute of Technology, Yokohama, Japan; ^6^ Brain Research Institute, Niigata University, Niigata, Japan; ^7^ Division of Human Genetics, Department of Integrated Genetics, National Institute of Genetics, Mishima, Japan; ^8^ Medical Genome Center, Research Institute, National Center for Geriatrics and Gerontology, Obu, Japan; ^9^ National Center for Child Health and Development, Tokyo, Japan

**Keywords:** RRAS2, Noonan-like phenotype, functional analysis, Ras/mapk signaling pathway, pathogenic variants, gain-of-function

## Abstract

**Introduction:** RRAS2, a member of the R-Ras subfamily of Ras-like low-molecular-weight GTPases, is considered to regulate cell proliferation and differentiation via the RAS/MAPK signaling pathway. Seven *RRAS2* pathogenic variants have been reported in patients with Noonan syndrome; however, few functional analyses have been conducted. Herein, we report two patients who presented with a Noonan-like phenotype with recurrent and novel *RRAS2* pathogenic variants (p.Gly23Val and p.Gly24Glu, respectively) and the results of their functional analysis.

**Materials and methods:** Wild-type (WT) and mutant *RRAS2* genes were transiently expressed in Human Embryonic Kidney293 cells. Expression of RRAS2 and phosphorylation of ERK1/2 were confirmed by Western blotting, and the RAS signaling pathway activity was measured using a reporter assay system with the serum response element-luciferase construct. WT and p.Gly23Val RRAS2 were expressed in *Drosophila* eye using the glass multiple reporter-Gal4 driver. Mutant mRNA microinjection into zebrafish embryos was performed, and the embryo jaws were observed.

**Results:** No obvious differences in the expression of proteins WT, p.Gly23Val, and p.Gly24Glu were observed. The luciferase reporter assay showed that the activity of p.Gly23Val was 2.45 ± 0.95-fold higher than WT, and p.Gly24Glu was 3.06 ± 1.35-fold higher than WT. For transgenic flies, the p.Gly23Val expression resulted in no adults flies emerging, indicating lethality. For mutant mRNA-injected zebrafish embryos, an oval shape and delayed jaw development were observed compared with WT mRNA-injected embryos. These indicated hyperactivity of the RAS signaling pathway.

**Discussion:** Recurrent and novel *RRAS2* variants that we reported showed increased *in vitro* or *in vivo* RAS signaling pathway activity because of gain-of-function *RRAS2* variants. Clinical features are similar to those previously reported, suggesting that *RRAS2* gain-of-function variants cause this disease in patients.

## 1 Introduction

The RAS/MAPK cascade is an important signaling pathway for the regulation of cell proliferation, differentiation, and survival. RASopathies, a collective term for diseases caused by abnormal signaling of the RAS/MAPK cascade, include Noonan syndrome (NS), Costello syndrome, cardiofaciocutaneous syndrome, neurofibromatosis type 1 and NS-like syndrome, and NS with multiple lentigines. It is challenging to differentiate these diseases because they share similar phenotypes that are specific to RASopathy, such as a distinctive facial appearance, cardiovascular abnormalities, musculoskeletal abnormalities, skin disorders, neurocognitive deficits, and an increased risk of tumors. Extensive genetic testing has been conducted, and new causative genes and pathogenic variants have been continuously discovered; thus, the disease spectrum is expanding.

RRAS2, a member of the R-Ras subfamily of Ras-like low-molecular-weight GTPases, is located in the plasma membrane and functions as a signal transducer. Like other RAS proteins, it is considered to regulate cell proliferation and differentiation via the RAS/MAPK cascade, and somatic mutations in the *RRAS2* gene are drivers of tumorigenesis; they have been detected in various tumors such as colon, lung, and skin cancers (Aoki et al., 2016; Clavaín et al., 2023). Furthermore, germline *RRAS2* mutations have been reported to cause NS (Capri et al., 2019; Niihori et al., 2019; Weinstock and Sadler, 2022; Yu et al., 2023). However, there are relatively few reports, and it is unclear what type of *RRAS2* variants cause the disease.

Herein, we report two patients with Noonan-like features who had undergone whole-exome sequencing (WES) analysis in the Rare and Undiagnosed Diseases Initiative Project (Takahashi and Mizusawa, 2021). We identified a recurrent (p.Gly23Val) and novel (p.Gly24Glu) *RRAS2* variant, which have not previously been functionally analyzed, in each patient with Noonan-like features. Furthermore, we report the *in vitro* and *in vivo* functions of these variants.

## 2 Materials and methods

### 2.1 Clinical presentation

**Case 1:** An 8-year-old boy was born at 39 weeks and 4 days; his weight was 4,292 g (+3.0 standard deviation [SD]). He had an anal atresia, a ventricular septal defect (VSD), and distinctive facial features (hypertelorism, down-slanted palpebral fissures, a broad nasal root, low-set ears, macrotia, and median facial depression). G-band karyotyping revealed a normal karyotype (46, XY). The VSD was small and closed spontaneously. He had no obvious developmental delays, had a WISC-IV score of 87 at age 6, and was enrolled in a regular elementary school. He was diagnosed with developmental coordination disorder; his height was 126 cm (+0.1 SD) at age 8, with no short stature. There were no abnormalities of the thorax, cryptorchidism, or lymphatic dysplasia.

**Case 2:** A 2-year and 10-month-old male with no family history of NS had one healthy brother. An ultrasound examination at 12 weeks of gestation revealed nuchal translucency. He was born at 37 weeks and 5 days of gestation via C-section; his weight was 4,682 g, and he had an Apgar score of 8/9/9. He had macrocephaly (+5.1 SD), distinctive facial features (high forehead, thick lower lip vermilion, hypertelorism, and low-set posteriorly rotated ears), bilateral cryptorchidism, grade 2 bilateral hydronephrosis, and hepatomegaly. G-band karyotyping was normal (46, XY), and an array-comparative genomic hybridization test showed no pathogenic copy number variations. Blood amino acid and organic acid analysis, newborn mass screening, and urinary uronic acid analysis were within normal ranges. He was able to hold his neck steady at 5 months, turn over at 5 months, sit up unaided at around 8 months, walk alone at 1 year and 9 months, and speak words at around 2 years of age. At 2 years and 10 months of age, he had a mild intellectual disability (his DQ was 68 on the Enjoji Scale of Infant Analytical Development Test); his height was 86 cm (−2.0 SD). There were no abnormalities of the thorax, congenital heart defect, or lymphatic dysplasia.

**FIGURE 1:**
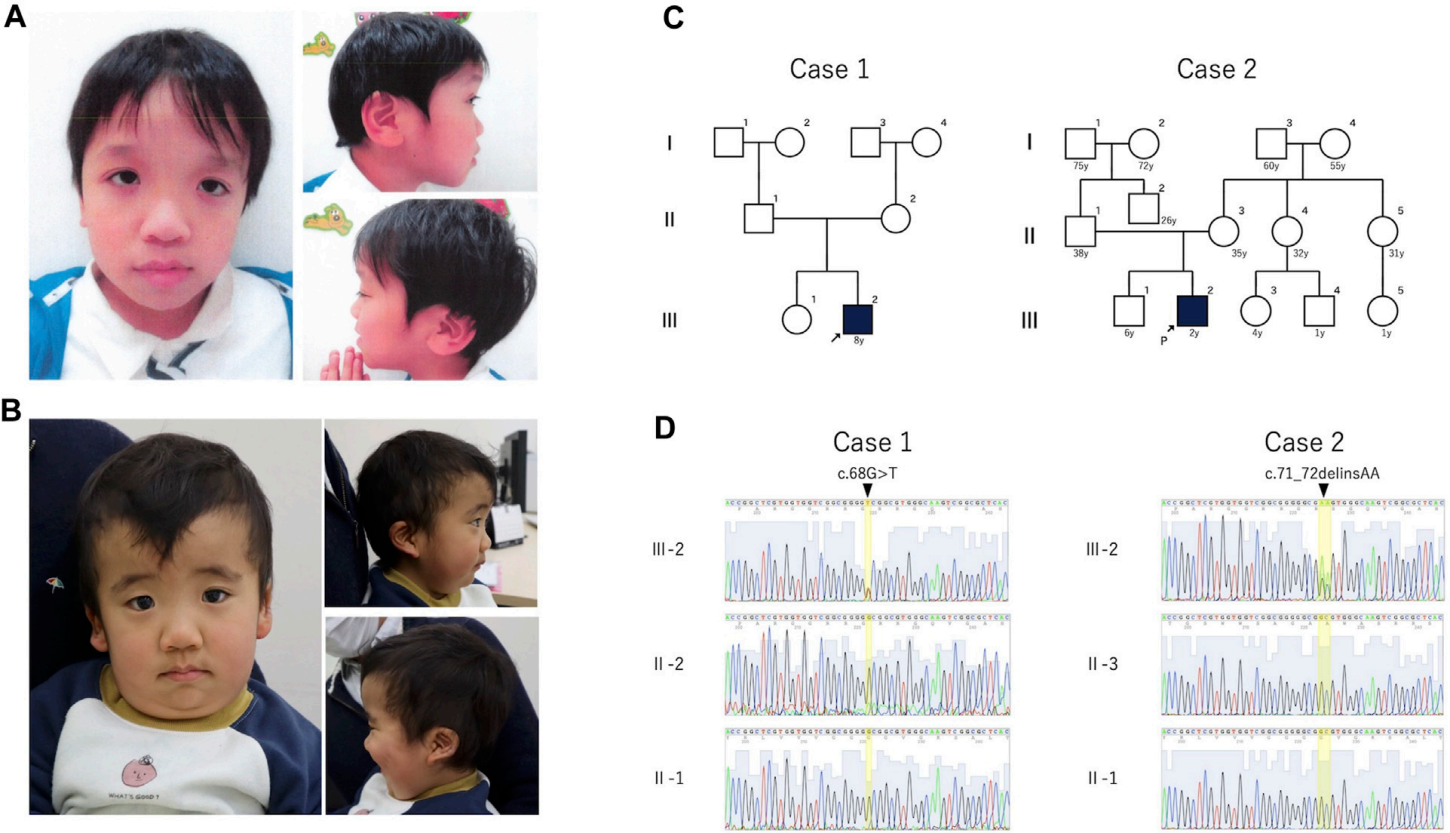
Photograph, family pedigree, and Sanger sequence results of the patients. **(A)**: Case 1 shows hypertelorism, down-slanted palpebral fissures, a broad nasal root, low-set ears, and macrotia. **(B)**: Case 2 shows hypertelorism, a high forehead, thick lower lip vermilion, and low-set posteriorly rotated ears. **(C)**: Family pedigrees of cases 1 and 2. Neither patient had a family history of these mutations. **(D)**: The **(C)**68G>T mutation was detected in case 1, and the c.71_72delinsAA variant was detected in case 2. Both variants were *de novo*.

The clinical features of the patients are presented in Table 1. Their facial photographs at the time of the most recent clinic visit are shown in Figures 1A,B, and their family trees are shown in Figure 1C.

**TABLE 1:** The clinical features of the patients.

	Case 1	Case 2
Age	8y	2y10m
Sex	Male	Male
*RRAS2* variant	c.68G>T	c.71_72delinsAA
RRAS2 protein change	p.Gly23Val	p.Gly24Glu
Prenatal features	-	cystic hygroma
Birth measurements		
weeks GA	39w4d	37w5d
weight(g)	4292 (+3.0 SD)	4692 (+5.1 SD)
length (cm)	-	52.1 (+2.3 SD)
OFC (cm)	-	39.4 (+5.1 SD)
Feeding difficulties	N	N
Height at last examination (cm)	126 cm, (+0.1 SD)	86 (-2.0 SD)
weight at last examination (kg)	28 kg, (+0.6 SD)	15.05 (+1.2SD)
OFC at last examination (cm)	-	52.5 (+2.0 SD)
Cryptorchidism	N	+, bilateral
Congenital heart defect	Ventricular septal defect	N
Lymphatic anomalies	N	N
Facial anomalies	dysmorphic features: hypertelorism, downslanted palpebral fissures, broad nasal root, low-set ears, macrotia, and median facial depression	dysmorphic features: high forehead, thick lower lip vermilion, hypertelorism, low-set posteriorly rotated ears
Pectus abnormalities	N	N
Development	WISC-Ⅳ 87, Regular class	intellectual disability, mild
Neurology	developmental coordination disorder, arachnoid cysts in the left middle cranial fossa	diffuse white matter lesions
Skeltal	N	Pes planus
Hematology and oncology	N	N
Ocular	Hypermetropia	Strabismus
Other malformations/anomalies	anal atresia, hearing impairment, broad thumb	thickened nuchal skin fold, bilateral hydronephrosis grade 2, hepatomegaly

### 2.2 Genetic analysis

After obtaining informed consent from the patients’ parents, we performed WES and filtering analysis in trio as previously described (Takeuchi et al., 2023). In brief, the SureSelect Human All Exon V6 Kit (Agilent Technologies, Santa Clara, CA, United States) was used for capturing, and a NovaSeq 6000 platform (Illumina, San Diego, CA, United States) was used for sequencing. CompStor Novos^®^ (Omnitier, San Jose, CA, United States) was used for secondary analysis and CompStor Insight^®^ (Omnitier) for tertiary analysis. GRCh38 and hg38 were used as the reference genomes.

The identified variants were confirmed by Sanger sequencing in the patients and their parents. The primers used for sequencing are listed in Supplementary Table S1.

### 2.3 *In vitro* functional analysis

The wild-type (WT) and variants of the *RRAS2* gene were cloned into expression vectors and transfected into Human Embryonic Kidney (HEK)293 cells for expression. Western blotting confirmed the RRAS2 expression and ERK1/2 phosphorylation. ERK exists downstream of the RAS/MAPK pathway and activates the Elk1 transcription factor in the nucleus, which binds to the serum response element (SRE) in complex with the serum response factor to promote gene expression. Phosphorylation of ERK1/2, the active form of ERK, suggested the activation of the RAS signaling pathway. The RAS signaling pathway activity was indirectly measured and quantified using luciferase activity with the co-transfection of a luciferase reporter vector that incorporated the SRE.

The primers used in these experiments are listed in Supplementary Table S1. In principle, we followed the previously reported experimental procedures by Niihori et al. (2019).

#### 2.3.1 Expression vector

*RRAS2* (NM_012250.6) cDNA was amplified using PCR with a cDNA library generated by the reverse transcription of the Takara total RNA master panel for human skeletal muscle RNA (Takara Bio Inc., Shiga, Japan). BamHI and EcoRI restriction enzyme sites were added to the forward and reverse primers, respectively. For the *RRAS2* (WT) expression vector, the PCR product was subcloned into the pcDNA™ 3.1 (+) Mammalian Expression Vector (Invitrogen, Carlsbad, CA) using the BamHI and EcoRI restriction sites. The constructs were confirmed by Sanger sequencing.

Next, the *RRAS2* variants (p.Gly23Val and p.Gly24Glu) were introduced respectively into the *RRAS2* WT expression vector via mutagenesis using the TaKaRa PrimeSTAR^®^ Mutagenesis Basal Kit (Takara). Furthermore, these were confirmed via Sanger sequencing.

#### 2.3.2 Western blotting

HEK293 cells were cultured in Eagle’s Minimal Essential Medium (MEM) containing 10% FBS and 1% penicillin/streptomycin (P/S) and adjusted to 1.0 × 10^5^ cells/mL. Five milliliters of this solution were dispensed in T25 flasks and incubated at 37°C for 24 h. The following day, 5 μg of pcDNA3.1 (+), the expression vector of WT, p.Gly23Val, or p.Gly24Glu, were transfected into the HEK293 cells using Lipofectamine^®^ 3000 Reagent (Thermo Fisher Scientific, Waltham, MA). Next, the cells were cultured for 48 h and the protein was extracted using RIPA buffer. Western blotting was performed according to the standard protocol using Anti–RRAS2 Polyclonal Antibody (#PA5-22123, Invitrogen), Phospho-p44/42 MAPK (Erk1/2) (Thr202/Tyr204) Antibody (#9101, Cell Signaling Technology, Danvers, MA, United States)), p44/42 MAPK (Erk1/2) Antibody (#9102, Cell Signaling Technology, Danvers, MA, United States)) and Anti–GAPDH (14C10) Rabbit mAb (#2118, Cell Signaling Technology, Danvers, MA, United States) as the primary antibodies. Anti–rabbit IgG, HRP-linked Antibody (#7074, Cell Signaling Technology) was used as the secondary antibody.

#### 2.3.3 Luciferase reporter assay

HEK293 cells were cultured in Eagle’s MEM containing 10% FBS with 1% P/S and seeded in 96-well plates at a density of 1.0 × 10^4^ cells/well. Following incubation at 37°C for 24 h, Lipofectamine^®^ 3000 Reagent was used to cotransfect the three vectors (expression, reporter, and *Renilla* control vector). *Renilla* control vector was used as an internal normalization standard to correct the transfection efficiency. For transfection, 1 μg of the expression vector plasmids (WT, p.Gly23Val, or p.Gly24Glu) or pcDNA 3.1 (+)were used as controls. The reporter vector used 100 ng of pGL4.33 [luc2P/SRE/Hygro] (Promega, Madison, WI), and the *Renilla* control vector used 10 ng of pRL *Renilla* Luciferase Control Reporter Vector (pRL-TK, Promega). Following incubation for 24 h, the culture medium was replaced with Eagle’s MEM containing 0.5% FBS and incubated for another 24 h. Luciferase activity was measured using the Dual-Glo^®^ Luciferase Assay System (Promega). We conducted the experiments according to the standard protocol and calculated the relative luciferase activity normalized to the *Renilla* luminescence. Each assay was performed in three separate wells and assayed six times independently.

### 2.4 Functional analysis in transgenic *Drosophila*

To evaluate the effects of the p.Gly23Val variant in RRAS2 *in vivo*, we generated transgenic flies to express either the reference or the p.Gly23Val variant of human RRAS2 in a tissue-specific manner. Both the RRAS2 reference and the p.Gly23Val variant were inserted into the same position in the genome using the phiC31 integrase system (Groth et al., 2004), ensuring no positional effects on gene expression. we expressed each variant in the *Drosophila* eye using the glass multiple reporter (GMR)-Gal4 driver (Newsome et al., 2000) to compare the functions of the WT RRAS2 and the p.Gly23Val variant.

#### 2.4.1 Fly strains

Flies were maintained at 25°C on standard fly food. GMR-Gal4 (#1104) was obtained from the Bloomington Stock Center.

#### 2.4.2 Generation of fly line for expression of human RRAS2 and the variant

To express human RRAS2 fused with an HA tag at the N-terminus, the sequences of HA-RRAS2WT and HA-RRAS2G23V were inserted into pJFRC81-10XUAS-IVS-Syn21-GFP-p10 (catalog no. 36432; Addgene) using primer 1 and primer 2 after removal of the GFP sequence. Details on primers were showed in Supplementary Table S1. The p.Gly23Val variant in *RRAS2* was generated using the overlap PCR technique with KOD One^®^ PCR Master Mix (TOYOBO, Osaka, Japan). To confirm the entire open reading frame sequence, bidirectional sequencing was performed using the Sanger method. These plasmids were injected into embryos and integrated into the attP40 landing site (WellGenetics, Taipei, Taiwan).

#### 2.4.3 Eye imaging using bright-field microscope

For light microscope imaging of adult fly eyes, 1-day-old flies, reared at 29°C, 25°C or 18°C to express RRAS2 WT via GMR-Gal4, was immobilized by freezing at −80°C. Subsequently, the flies were mounted on a microscope slide using double-sided tape. The eyes of the flies were imaged using a BX53 microscope system equipped with a MPLFLN ×20 objective lens (Olympus, Tokyo, Japan). The phenotypic scores were calculated using Flynotyper (Iyer et al., 2016).

### 2.5 Functional analysis of zebrafish embryos

Total RNA was extracted from ARPE-19 cells using the RNeasy mini kit (Qiagen, Hilden, Germany) and reverse transcribed to generate cDNA using the ReverTra Ace kit (TOYOBO Co., Ltd., Osaka, Japan). The *RRAS2* coding sequence (CDS) was amplified by PCR, and the amplified CDS was cloned into a pCS2+ vector using the iVEC3 system (Nozaki and Niki, 2019). The sequence was confirmed by Sanger sequencing, and the p.Gly23Val and p.Gly24_Gly26dup mutations were introduced using the TOYOBO KOD-Plus Mutagenesis Kit. The p.Gly24_Gly26dup mutation was a previously reported mutation (Niihori et al., 2019) and was used as a positive control. Three mRNAs (WT, p.Gly23Val, and p.Gly24_Gly26dup) were generated using the mMessage mMachine SP6 Transcription kit (Invitrogen). The mRNAs were purified using the RNeasy kit (Qiagen) and microinjected into fertilized zebrafish ova at 25 pg each using FemtoJet 4i (Eppendorf, Hamburg, Germany). The anterior-posterior length and dorsal-ventral length were measured at 12 h post-injection, and their ratios (AP/DV ratio) were calculated. The four-day-old embryos were fixed with 4% paraformaldehyde (PFA), stained with Alcian blue, and photographed. The jaw cartilage angle was measured using ImageJ2 software version 2.3.0. The primers used in the experiments are listed in Supplementary Table S1. These experimental procedures were performed in accordance with previously reported procedures (Niihori et al., 2019).

### 2.6 Statistical analysis

Statistical analyses were performed using the multicomp package in R Studio version 4.3.1. The relative luciferase activity in the luciferase assay was calculated as the mean value from the three wells for each assay, and the fold change was calculated against that of the WT. Dunnett’s test was used for the statistical test of the relative luciferase activity, AP/DV ratio, and jaw cartilage angle in the zebrafish embryos. A two-tailed *p*-value, 0.05 was regarded as significant.

## 3 Results

### 3.1 Genetic analysis

In cases 1 and 2, we identified a *de novo* heterozygous variants of *RRAS2*, NM_012250.6:c.68G>T, p.Gly23Val and *RRAS2*, NM_012250.6: c.71_72delinsAA, p.Gly24Glu, respectively (Figure 1D). The candidate variants detected by WES analysis in both patients were listed in Supplementary Table S2.

### 3.2 Western blotting

**FIGURE 2:**
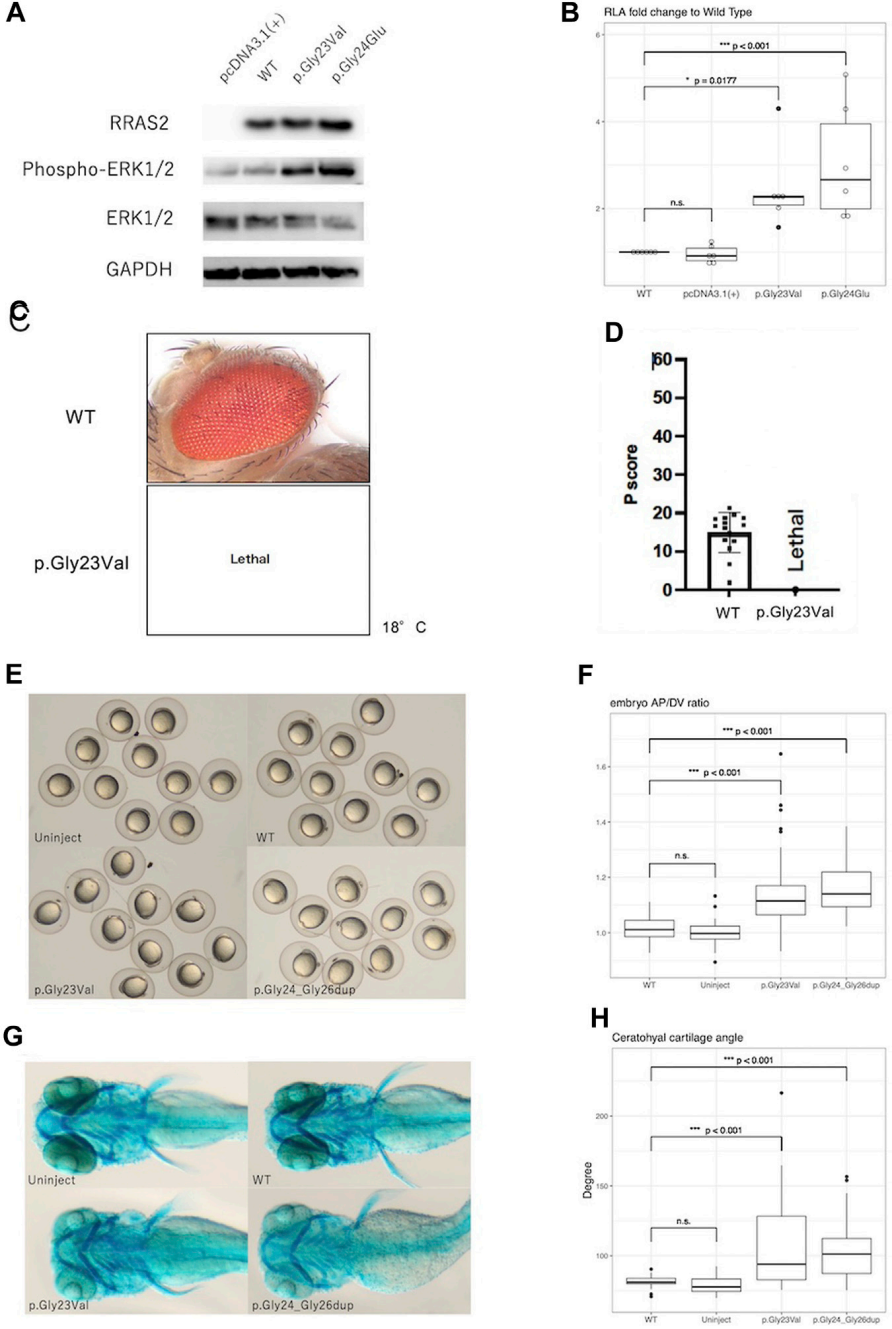
Results of functional analysis experiments. **(A)**: Western blotting detected the expression of RRAS2 protein and phosphorylation of ERK1/2 protein. RRAS2 had no differences in size were observed between the wild-type (WT), p.Gly23Val, and p.Gly24Glu. The levels of ERK1/2 phosphorylation were increased in p.Gly23Val and p.Gly24Glu compared to wild type. **(B)**: The fold change in the relative luciferase activity of the p.Gly23Val and p.Gly24Glu constructs against that of the WT is shown. pcDNA3.1 (+) was not significantly different, whereas p.Gly23Val and p.Gly24Glu were both significantly elevated (*n* = 6). **(C,D):** The light microscope images of the fly eyes expressing WT RRAS2 and p.Gly23Val RRAS2 and the phenotypic scores of them calculated by Flynotyper. WT showed normal phenotype and p.Gly23Val was lethal. **(E)**, **(F)**: Zebrafish embryos at 12 h after mRNA injection. p.Gly23Val and p.Gly24_Gly26dup (positive control) embryos were oval in shape, although the WT and uninjected embryos were circular **(E)**. The AP/DV ratios were significantly larger in p. Gly23Val and p.Gly24_Gly26dup than in the WT **(F)**. The number of embryos observed and measured was 83 in the WT, 60 in the uninjected, 92 in the p.Gly23Val, and 43 in the p.Gly24_Gly26dup. **(G)**, **(H)**: Zebrafish 4-day embryos after mRNA injection. Compared with the WT and uninjected embryos, the jaw cartilage angles were larger in p.Gly23Val and p.Gly24_Gly26dup **(G)**, which were statistically significant **(H)**. The number of 4-day embryos observed and measured was 32 in the WT, 30 in the uninjected, 34 in the p.Gly23Val, and 38 in the p.Gly24_Gly26dup.

Western blot analysis showed no obvious differences in expression of WT, p.Gly23Val, and p.Gly24Glu proteins. In contrast, phosphorylation of ERK1/2 was elevated in p.Gly23Val and p.Gly24Glu compared to the wild type (Figure 2A).

### 3.3 Luciferase reporter assay

In the luciferase reporter assay, the fold change of the relative activity of pcDNA3.1 (+), p.Gly23Val, and p.Gly24Glu to that of the WT was 0.95 ± 0.20, 2.45 ± 0.95, and 3.06 ± 1.35, respectively (mean ± SD, Figure 2B). Both variants exhibited significantly increased activity compared with that of the WT.

### 3.4 Evaluation of pathogenicity of RRAS2 variant in transgenic *Drosophila*

Expression of the WT RRAS2 had no effect on the retina. However, expressing the p.Gly23Val RRAS2 transgene resulted in no adult flies emerging even at 18°C, indicating lethality (Figures 2C, D). Since *Drosophila* can survive even without their eyes, the GMR-Gal4 driver is often used to study the mutational effects of disease-related genes. Nevertheless, the lethality observed in these flies suggests that p.Gly23Val, which was expressed in very small amounts in tissues outside of the eye, may be highly toxic. This result suggests that p.Gly23Val may have a gain of toxic function.

### 3.5 Zebrafish embryo analysis

After 24 h, the embryos injected with p.Gly23Val mRNA exhibited an oval shape (Figure 2E). The AP/DV ratios of the WT and p.Gly23Val mRNA-injected embryos were 1.014 ± 0.04 and 1.129 ± 0.112, respectively. The AP/DV ratio of p.Gly23Val mRNA-injected embryos was substantially elevated compared with that of the WT (Figure 2F). The 4-day-old p.Gly23Val mRNA-injection embryos exhibited an enlarged jaw cartilage angle and delayed jaw development (Figure 2G). The jaw cartilage angles of the WT and p.Gly23Val mRNA-injection embryos were 81.66 ± 4.04 and 108.4 ± 32.66, respectively. The jaw cartilage angle in the p.Gly23Val mRNA-injected embryos was considerably larger than that of the WT (Figure 2H).

## 4 Discussion

In this study, we reported two patients with Noonan syndrome-like clinical features and a recurrent and novel *RRAS2* pathogenic variant, p.Gly23Val and p.Gly24Glu, respectively. Furthermore, we experimentally demonstrated that both these variants were gain-of-function variants that activated the RAS signaling pathway. The phosphorylation levels of ERK1/2 and the reporter assay results suggested increased activity of the RAS signaling pathway, and the introduction of the p.Gly23Val variant revealed a gain of toxicity in *Drosophila* and an increased AP/DV ratio and angle of the jaw cartilage in zebrafish embryos. The p.Gly24Glu is novel and we first reported the results of functional analysis *in vitro*. We also presented the results of functional analysis of p.Gly23Val *in vivo* (in zebrafish and *Drosophila*), which has not been reported previously.

Reports of NS caused by *RRAS2* are relatively recent; however, there are few reports on their functional analysis. A previous report indicated that the activation of the RAS pathway by a gain-of-function mutation in the *RRAS2* gene caused the disease and demonstrated an increase in the RAS signaling pathway (Niihori et al., 2019). Our data support these findings. Although we were unable to perform experiments on the p.Gly24Glu mutant in zebrafish and *Drosophila*, we believe that the results of *in vitro* experiments provided evidence for the determination of gain-of-function variant, considering the previous reports and functional analysis of RAS-related proteins other than RRAS2 (Kobayashi et al., 2010; Aoki et al., 2013; Higgins et al., 2017).

No adult flies emerged at 29°C and 25°C, and even at 18°C. It was surprising that *Drosophila* was lethal even at 18°C, because GAL4 activity depended on temperature (Duffy, 2002). These results suggest that the expression of p.Gly23Val may have significant effects in *Drosophila*.

**FIGURE 3:**
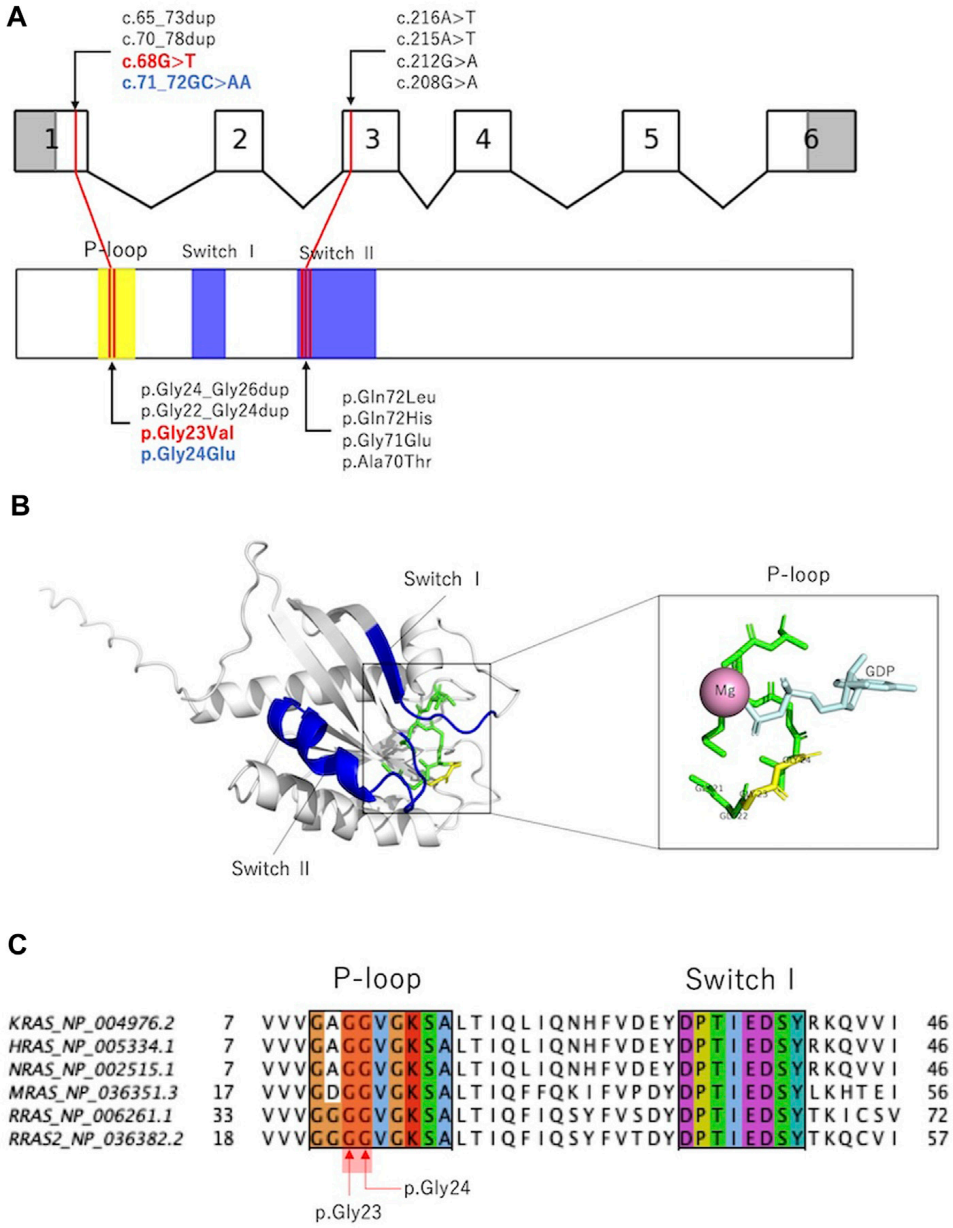
Previously reported *RRAS* variants and their *in silico* analysis. **(A)**: Exon–intron map of RRAS2 and functional domains of the RRAS2 proteins with their reported variants. **(B)**: Structure of the human RRAS2. The entire phosphate-binding loop is shown in green, and p.Gly23 and p.Gly24 are shown in yellow. Structural modeling was performed using PyMOL based on RRAS2 (PDB ID: 2ERY). **(C)**: Partial amino acid sequence alignments of human KRAS, HRAS, NRAS, MRAS, RRAS, and RRAS2. p.Gly23 and p.Gly24 were observed in the phosphate-binding loop. Alignments were generated using MAFFT version 7 online.

RAS is activated by the conversion of GDP-bound to GTP-bound forms and regulates downstream signal transduction. The RAS molecule has two important domains: the phosphate-binding loop that binds to nucleotide β-phosphate, and Switch I and II, which undergo conformational changes during the hydrolysis reaction from GTP to GDP (Simanshu et al., 2017). Previously reported variants were localized to the phosphate-binding loop and Switch II domains (Capri et al., 2019; Niihori et al., 2019; Weinstock and Sadler, 2022; Yu et al., 2023), and the variants in the present cases were located in the phosphate-binding loop (Figures 3A,B). p.Gly24Glu is a novel variant, and *in silico* predictions revealed a SIFT score of <0.05, which is calculated deleterious, and a polyphen2 score of 1.00, which is calculated probably damaging. These deleterious predictions were inferred from the substituted amino acid, which is an acidic (polar) Glu from a nonpolar Gly.

Figure 3C and Supplementary Table S3 show amino acid variants at the homologous sites of proteins that are homologous to RRAS2 (KRAS, HRAS, NRAS, MRAS, and RRAS). All variants are registered as pathogenic in ClinVar, and the variants that are homologous to p.Gly23Val have been reported to cause RASopathy in KRAS, HRAS, and NRAS. Interestingly, among the amino acid mutations that are homologous to the 24th amino acid in RRAS2, the substitution for glutamic acid was not registered, although substitutions for nonglutamic acid are registered as pathogenic.

These findings suggest that the identified *RRAS2* pathogenic variants enhanced the activity of the RAS signaling pathway, which led to the development of the disease. Our report provides further insight into the mechanism via which pathogenic variants of RRAS2 cause NS. Further accumulation of cases such as these will reveal increased detailed molecular pathology.

## Data Availability

The original contributions presented in the study are included in the article/Supplementary Material, further inquiries can be directed to the corresponding author. The WES sequence data of the patients are not publicly available due to their containing information that could compromise the privacy of research participants. Further inquiries can be directed to the corresponding author/s.
